# Supplementary material for: Structural insights into human topoisomerase 3β DNA and RNA catalysis and nucleic acid gate dynamics
Source: Nat Commun. 2025 Jan 19;16:834. doi: 10.1038/s41467-025-55959-y (PMC11743793; doi:10.1038/s41467-025-55959-y)
Supplement: Supplementary file 1 — Supplementary Information [file 41467_2025_55959_MOESM1_ESM.pdf]

Supplementary Information

Structural insights into human Topoisomerase 3β DNA and RNA catalysis and nucleic acid gate dynamics

Xi Yang[1], Xuemin Chen[2,3], Wei Yang[2]✉ & Yves Pommier[1]✉

[1]Developmental Therapeutics Branch & Laboratory of Molecular Pharmacology, Center for Cancer Research, National Cancer Institute, NIH, Bethesda, MD 20892, USA. [2]Laboratory of Molecular Biology, National Institute of Diabetes and Digestive and Kidney Diseases, NIH, Bethesda, MD 20892, USA. [3]School of Life Sciences, Anhui University, Hefei, China.

✉e-mail: pommier@nih.gov or weiy@niddk.nih.gov.

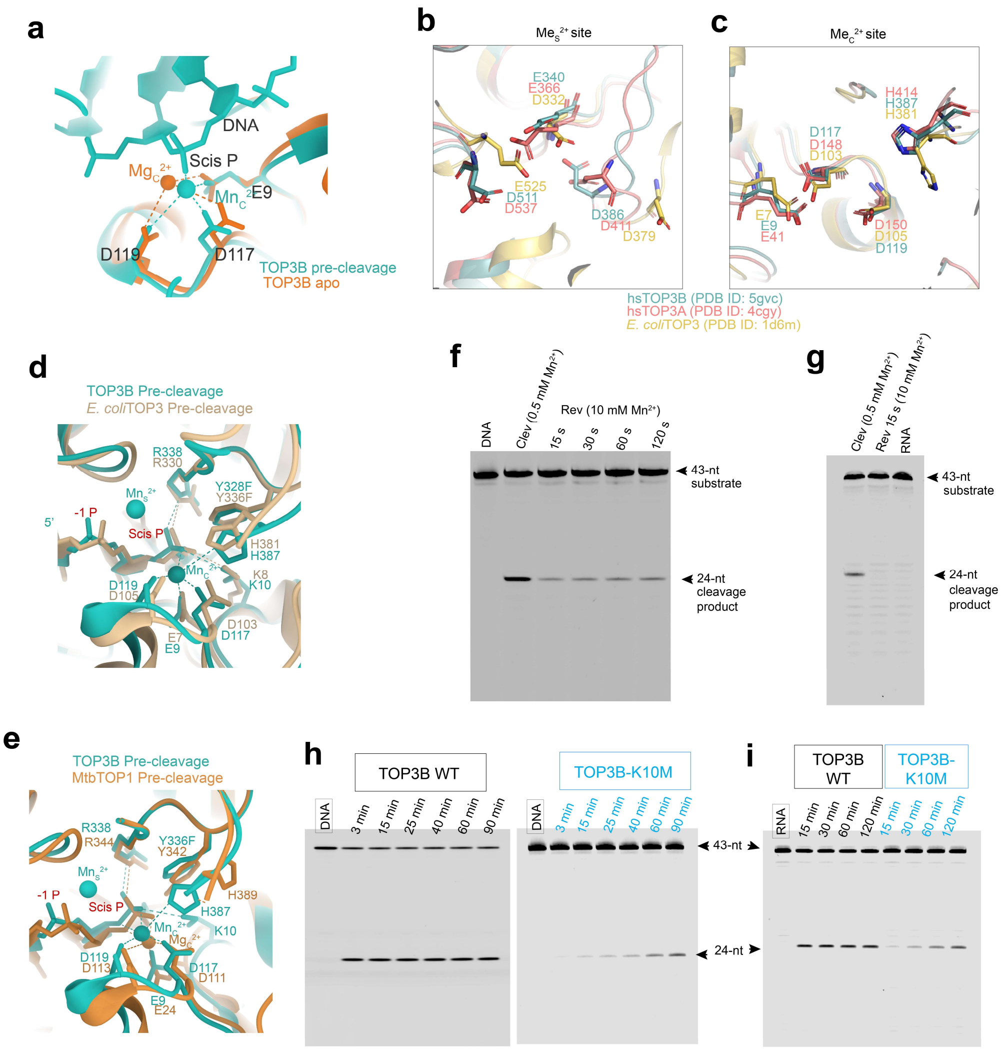

Supplementary Fig. 1: Active-site residues and divalent-cation (Me^{2+}) binding sites across TopoIA family.

a, Superposition of the apo (PDB ID: 5gve) and DNA-bound TOP3Bcore. Dotted lines represent Mn^{2+}/Mg^{2+} coordination. **b**,**c**, Superimposed metal-ion binding sites of the structural and catalytic divalent cations (Me_S^{2+} and Me_C^{2+}) in human TOP3B (cyan), TOP3A (pink) and *E.coli*TOP3 (yellow). The conserved metal-ion coordinating residues are highlighted. **d**, Superimposed pre-cleavage state active sites of TOP3B and *E.coli*TOP3 (PDB ID: 1i7d). The former contains two divalent metal ions Me_C^{2+} and Me_S^{2+} while the latter structure lacks divalent ions, likely due to the protonated carboxylates and phosphate at the pH 5.5 crystallization conditions. **e**, Superposition of TOP3B and MtbTOP1 (PDB ID: 6cq2). Both enzymes have the catalytic metal ion Me_C^{2+} while the latter lacks the Me_S^{2+}. The conserved lysine residue in TOP3 enzymes is missing in MtbTOP1 and other TOP1 enzymes. Unlike H387 in TOP3B, the conserved Histidine (H389) in MtTOP1 does not serve as a ligand for Me_C^{2+}. **f**, DNA cleavage by TOP3B (at 0.5 mM Mn^{2+}) and time course of re-joining following addition of 10 mM Mn^{2+}. High Mn^{2+} concentration induces rapid reversal of DNA cleavage. DNA substrate and product bands are described in Fig. 1b,c. **g,** RNA cleavage and rejoining assay conducted under identical Mn^{2+} conditions as the DNA assay. All cleaved RNA was rapidly religated within 15 s after adding 10 mM Mn^{2+}. **h**,**i**, Time course of DNA (**h**) and RNA (**i**) cleavage by TOP3B and TOP3B-K10M. Substrate and cleavage products are described in Fig. 1b. Results shown in panel f, g, h and i are all representative results from at least two independent experimental replicates. Source data are provided as a Source Data file.

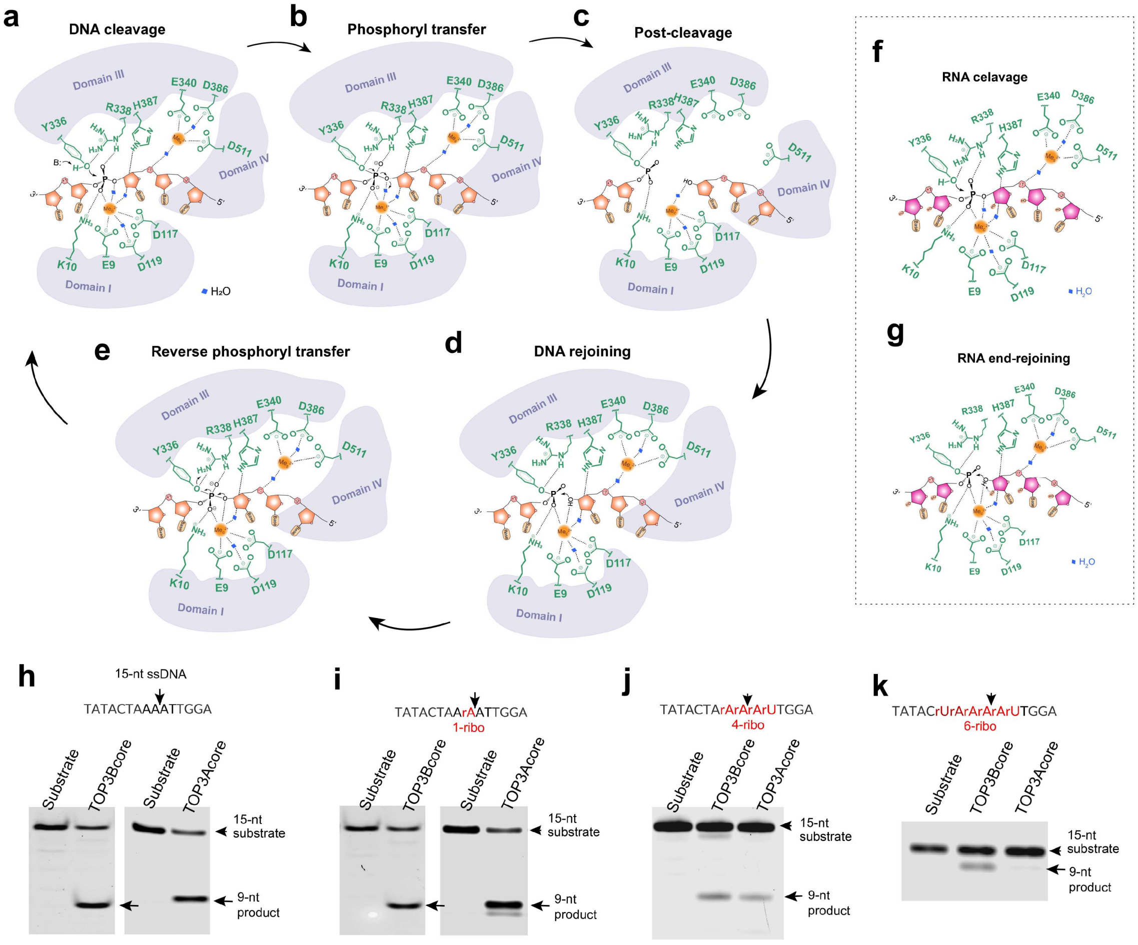

Supplementary Fig. 2: DNA and RNA catalysis by TOP3B, and comparison with TOP3A.

a, DNA pre-cleavage state. The catalytic metal ion (Me_C^{2+}) is coordinated by four residues E9, D117, D119 and H387. Me_C^{2+} together with K10 and R338 play a role in precisely positioning the scissile phosphate. The structural metal ion Me_S^{2+} is coordinated by three acidic residues and contributes to fine-tuning the position of the -1 phosphate group. Together these elements facilitate the initiation of the nucleophilic attack by Y336, potentially stimulated by a general base (B:). Blue diamond symbols indicate divalent metal-coordinated water molecules. **b**, Intermediate state of phosphoryl transfer. Me_C^{2+}, K10 and R338 serve to neutralize the highly negatively charged transition-state pentavalent phosphate upon nucleophilic attack and stabilize the 3'-oxygen leaving group. **c**, Following phosphoryl transfer, a tyrosyl-phosphate group is formed at the 5'-end of the cleaved DNA, remaining connected to K10 and R338. Me_C^{2+} is reserved and remains in contact with the 3'-OH group of the cleaved DNA, while Me_S^{2+} is absent after DNA cleavage. **d**, Initiation of DNA end-rejoining. Me_C^{2+} and Me_S^{2+} along with residues R338 and K10 properly position the tyrosyl phosphate and the 3'-OH group, facilitating the activation of a nucleophilic attack by the 3'-OH group (indicated by the arrow). **e**, Intermediate state of reverse phosphoryl transfer. Negative charges accumulated in the transition-state pentavalent phosphate are stabilized by Me_C^{2+}, R338, and K10. Arrows illustrate the electron flow from the nucleophile to the leaving group which is facilitated by the guanidino group of R338. **f**, Active-site reactions in RNA cleavage are similar to DNA as described in (**a**). **g,** RNA rejoining shares a comparable mechanism with DNA as illustrated in Figure S2D, except that the 2'-OH group in RNA near the nucleophile 3'-OH can polarize the nucleophile or readily act as a general base that accelerates the RNA rejoining process (indicated by arrows). **h,** TOP3B and TOP3A cleavage with a 15-nt ssDNA containing a shared cleavage site (arrow). **i,** TOP3B and TOP3A cleaving a 15-nt ssDNA derivative with a ribonucleotide at the cleavage site. **j**,**k**, TOP3B and TOP3A cleavage assays with substrates containing four ribonucleotides (two upstream of the cleavage site) and six ribonucleotides (four upstream). Results shown in panel h-k are all representative results from at least two independent experimental replicates. Source data are provided as a Source Data file.

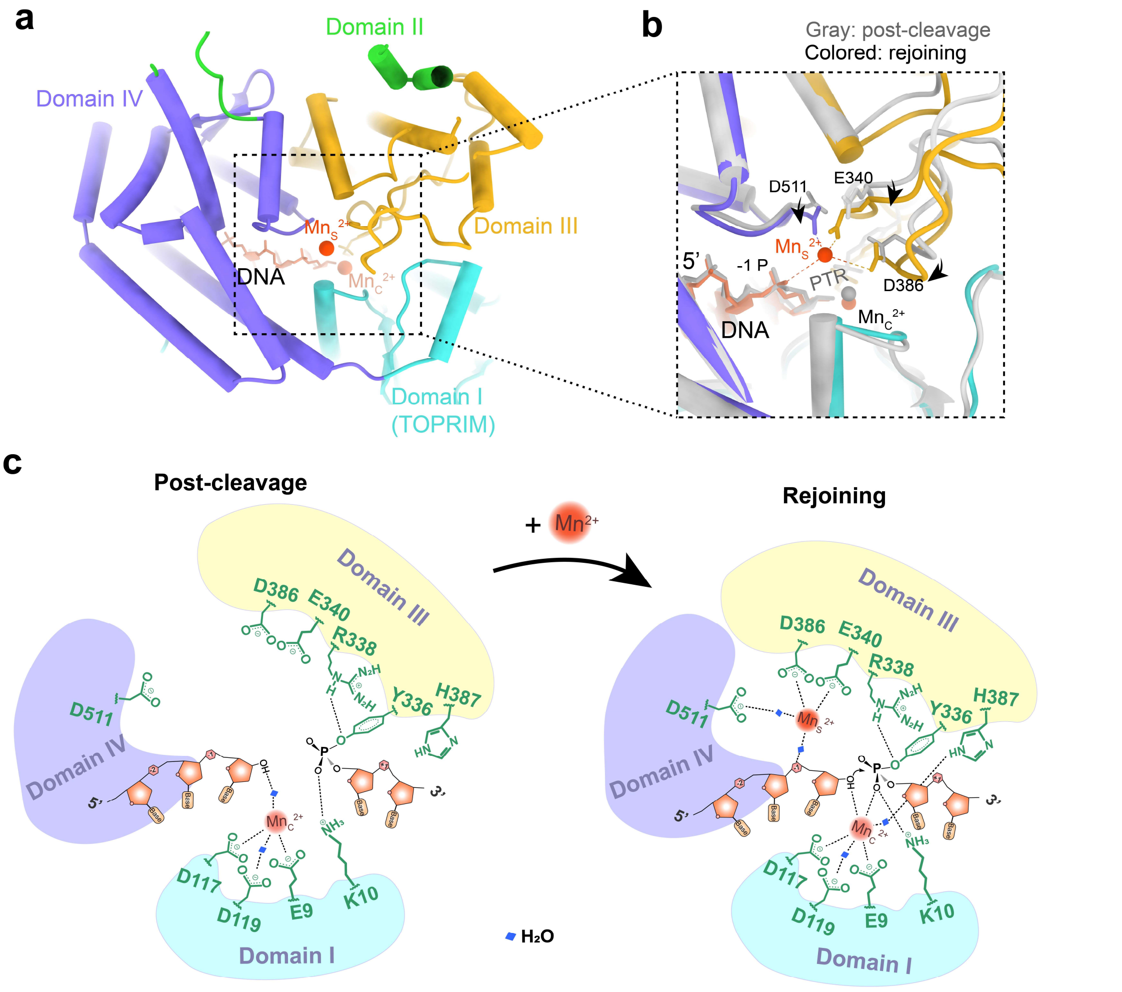

Supplementary Fig. 3: Mn$_S$$^{2+}$ binding promotes TOP3B domain convergence, crucial for DNA rejoining.

a, DNA rejoining state complex. The structural cation Mn$_S$$^{2+}$ (red) is located at the interface among domains I, III, and IV. **b**, Close-up view of the Mn$_S$$^{2+}$ binding site in a, superimposed with the DNA post-cleavage complex (grey). Mn$_S$$^{2+}$ binding stabilizes a relatively tight protein conformation. It brings domain III closer to domains I and IV via interaction with the highlighted acidic residues. PTR: phosphor-tyrosyl linkage. Dotted lines highlight Mn$_S$$^{2+}$ coordination. **c**, Mn$_S$$^{2+}$ binding brings domains I, III, and IV into proximity, ensuring optimal positioning of the DNA ends, the catalytic cation Mn$_C$$^{2+}$, and the catalytic residues for DNA rejoining.

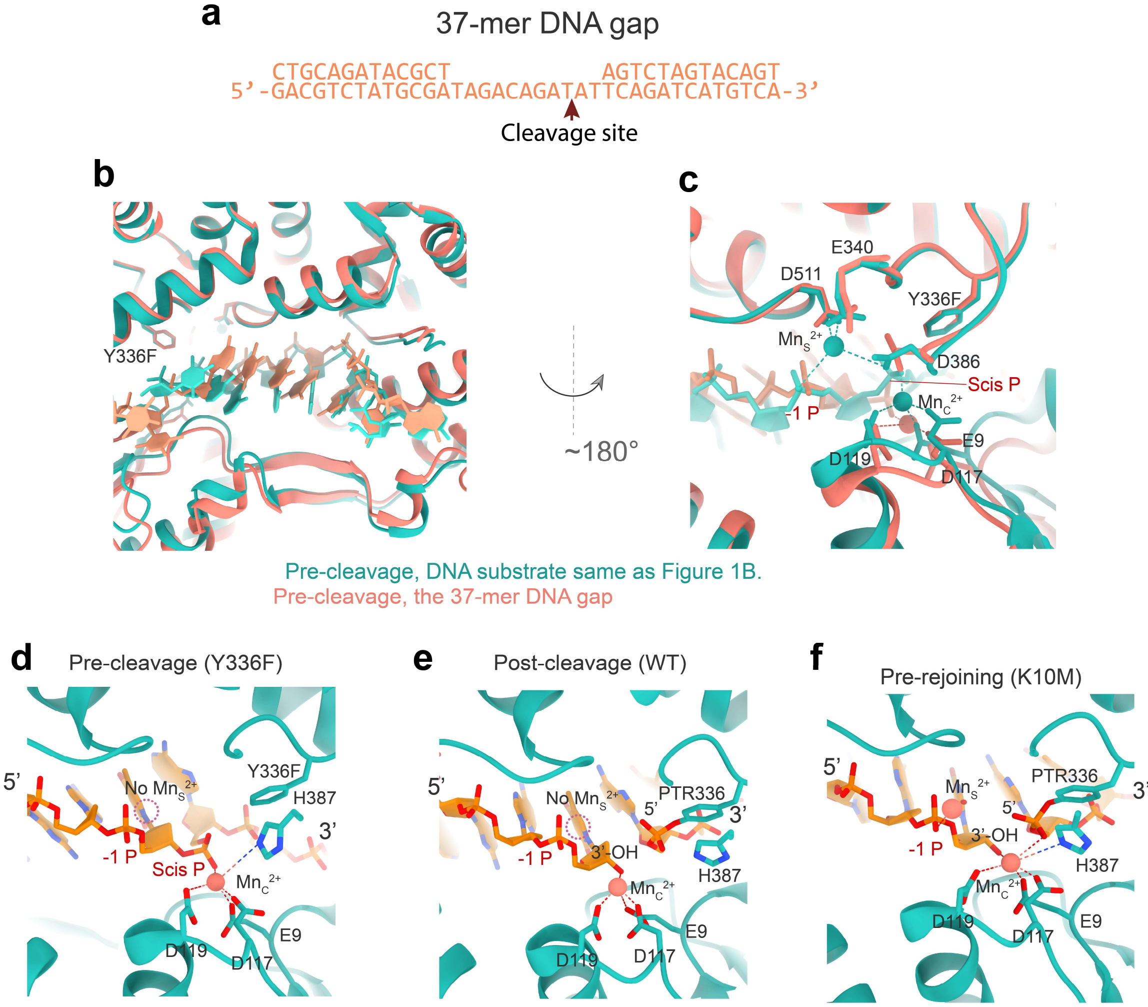

Supplementary Fig. 4: Active-site configurations and ssDNA binding conformation of cTOP3B with a different gapped DNA substrate.

a, 37-mer DNA gap substrate (Gap 2) containing a strong TOP3B cleavage site (indicated by arrow). **b**, Superposition of the two DNA fragments with different sequences inside the TOP3B DNA binding groove. **c**, Superposition of the active sites of the two DNA complexes described in (**b**). **d**, **e**, Catalytic-site configurations of the DNA pre-cleavage (Y336F mutant), post-cleavage (wild-type) and pre-rejoining (K10M mutant) complexes generated with the 37-mer DNA gap substrate. Mn_C^{2+} is missing at both the pre-cleavage and post-cleavage states, highlighted with dotted circles.

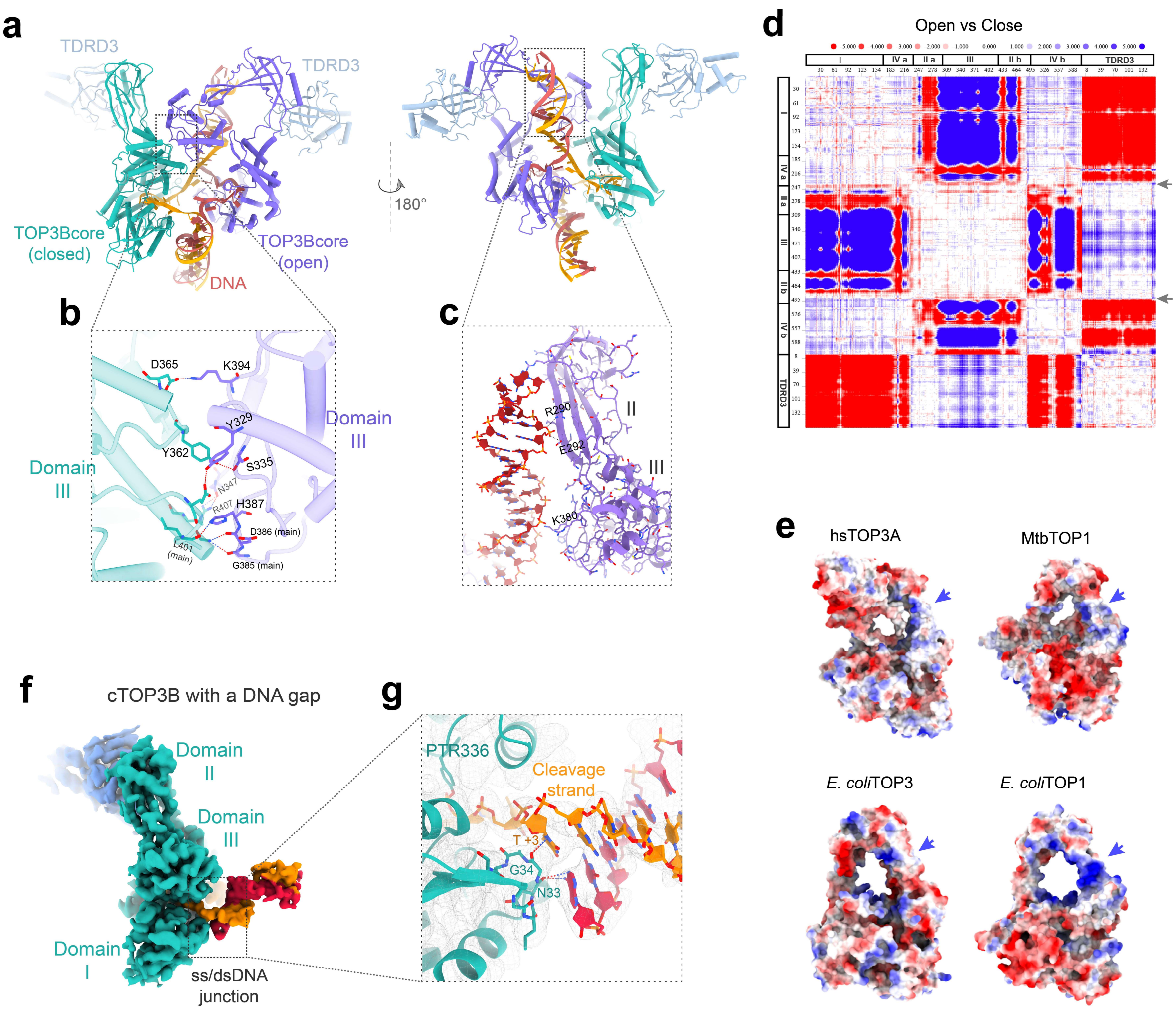

Supplementary Fig. 5: Structural analysis of the cTOP3B dimer, and comparison between the open and closed conformations of TOP3B.

a, Atomic model of the cTOP3B dimer as depicted in Fig. 4a. **b**, Interactions between domain IIIs of two cTOP3B molecules. "(main)" indicates main-chain interactions. **c**, Interactions of dsDNA with highlighted positively charged residues from domains III and II. **d,** DDMP (difference distance matrix plot) of cTOP3B, open versus closed conformations. Delta values between the difference distance matrixes of the two forms of cTOP3B are plotted with red and blue dots, indicating the Cα differences from -5 to 5 Angstroms (darker color shows larger differences). Numbers on top and at left indicate TOP3B and TDRD3 amino-acid positions. Domains are highlighted. The plot shows that domain III with a part of domain II rotates relative to domains I and IV. Arrows highlight hinge loop regions, delineating the boundaries of significant conformational changes. **e,** Electrostatic potential surfaces of TopoIA enzymes showing their conserved positive charge profile at the indicated areas of domains II and III. **f**, **g**, cTOP3B cleavage complex with a gapped DNA showing the position of the dsDNA arm and interactions with the enzyme in the closed state (same structure in Fig. 4g), and difference from those in the open state (Fig. 4h).

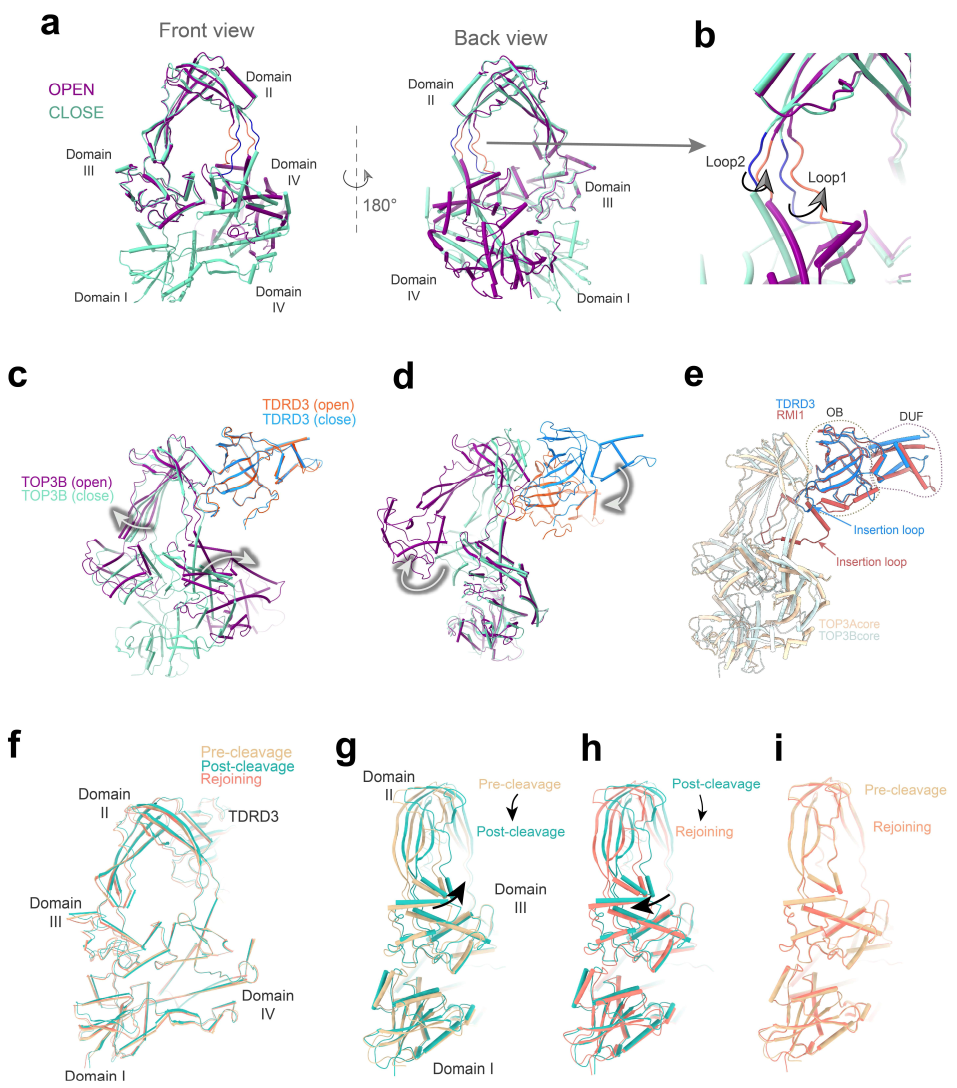

Supplementary Fig. 6: TOP3B Hinge loops and domain movements within a TOP3B catalytic cycle.

a, **b**, Aligning domains III and II of the open and closed TOP3Bcore (together with domains I and IV superimposed in Fig. 5c) demonstrates the hinge motion (arrows) of two loops connecting domains II and IV upon topo gate opening. **c,** Superposition of the N-terminal TDRD3 in the open and closed cTOP3B reveals an unchanged conformation of the DUF-OB fold of TDRD3 along with the upper portion of domain II of TOP3B upon topo-gate opening. Domains II-III and domains I-IV move in opposite directions relative to each other, also illustrated in Extended Data Fig. 5d. **d,** Superimposed open and closed cTOP3B showing the movement of the N-terminus of TDRD3 alongside domains II and III, upon TOP3B gate opening. **e,** The N-terminal DUF-OB folds of Rmi1 and TDRD3 share a similar shape, except in their variable insertion loops within their OB folds. **f,** Superposition of cTOP3B (domains I and IV) at three enzymatic states: pre-cleavage, post-cleavage and rejoining. **g-I,** Pairwise comparisons of three cTOP3B intermediates showing movements of domains III and II, whose directions are in accordance with gate opening and closure as described in Fig. 4c.

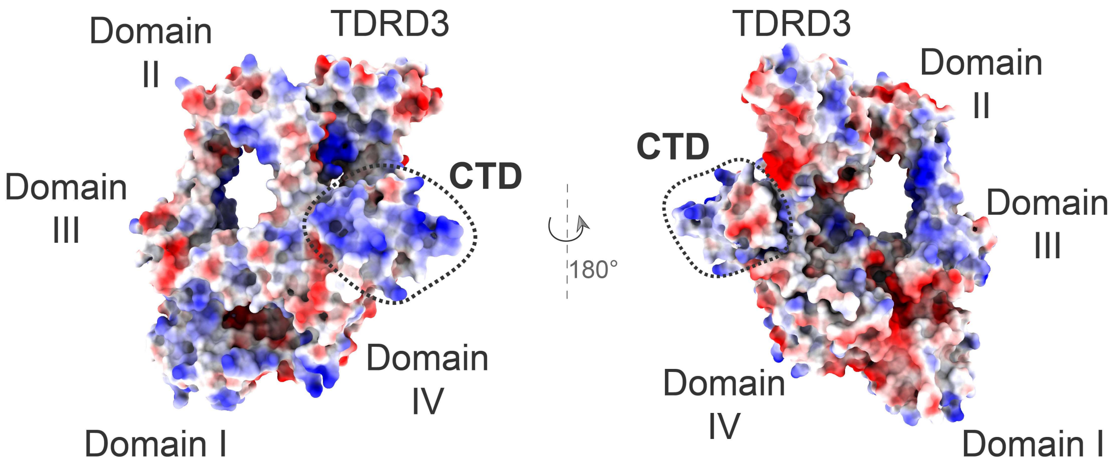

Supplementary Fig. 7: Electrostatic surface potential of TOP3B-TDRD3.

Electrostatic potential surfaces of TOP3B-TDRD3 generated with the atomic model in Fig. 5b.

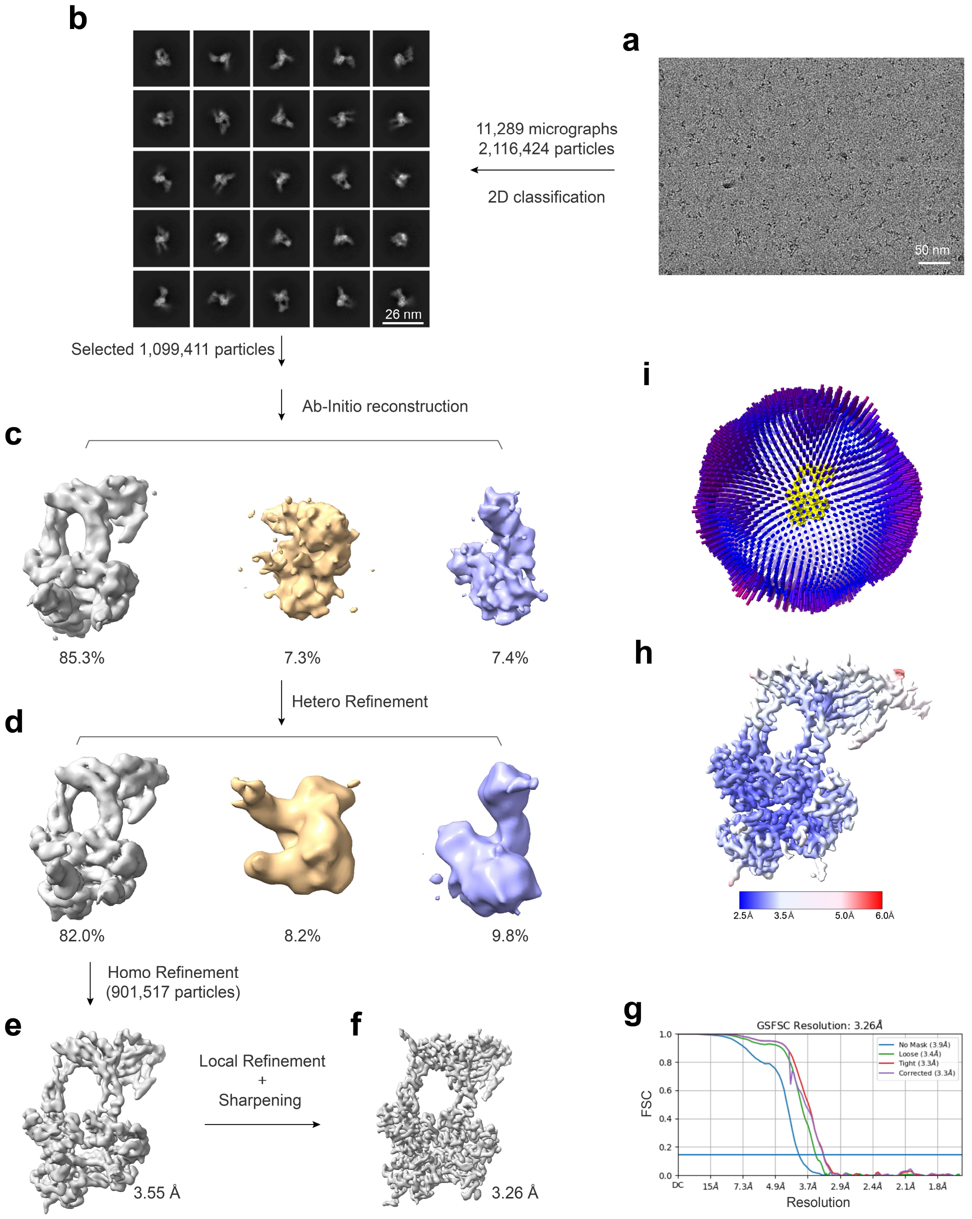

Supplementary Fig. 8: Cryo-EM Structure determination of DNA post-cleavage state TOP3B-TDRD3 core complex using cryoSPARC.

a-f, CryoEM data processing workflow, representing the workflows for all the cryo-EM maps in the manuscript. **g**, FSC curve with the threshold 0.143 indicated. **h**, cryo-EM map in panel f colored by local resolution with color scale bar. **i**, Angular distributions of particles aligned with the final reconstruction in yellow. Red cylinders represent orientations with more particles.

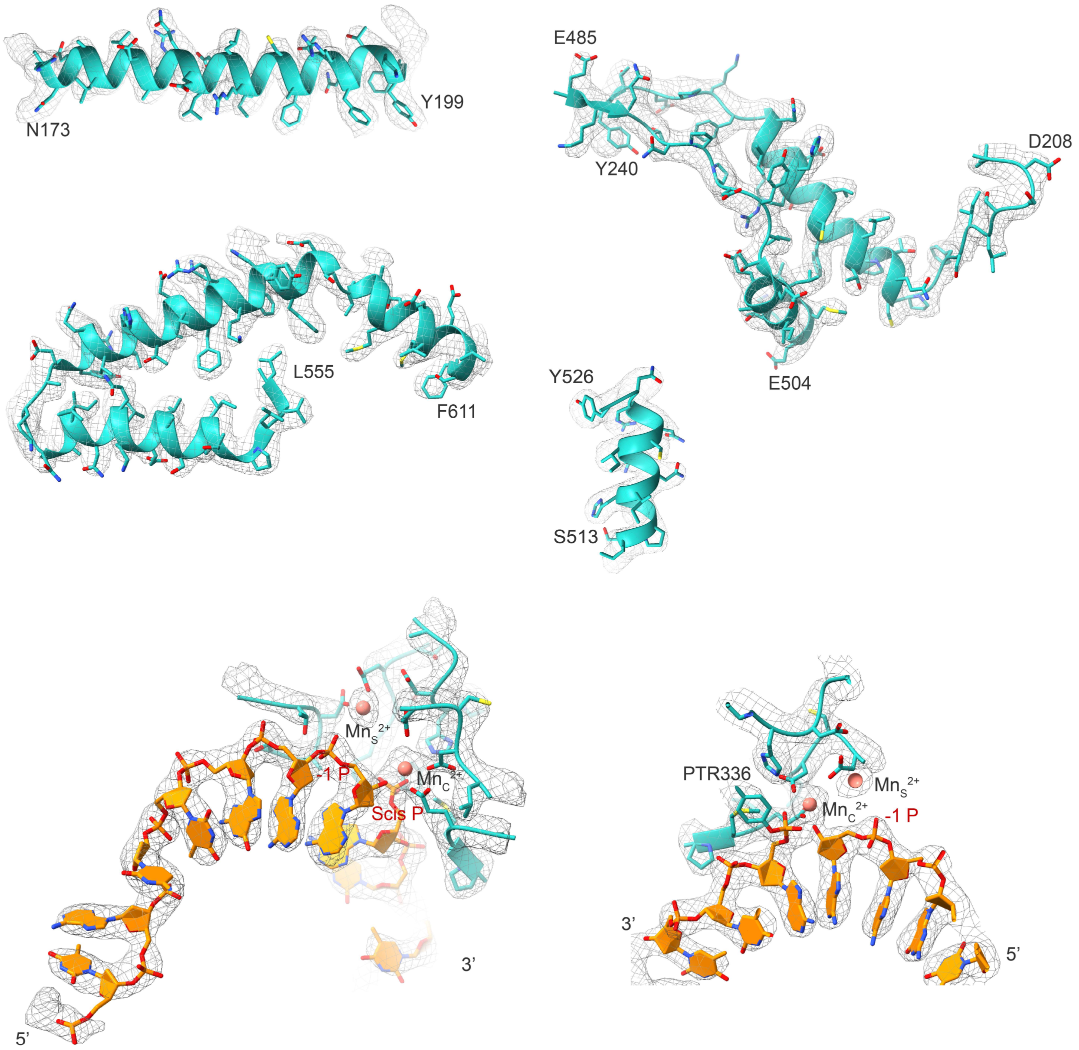

Supplementary Fig. 9: Representative map regions superimposed with model.

Representative regions of the final reconstructed cryo-EM map shown in Extended Data Fig. 8f (grey mesh), superimposed with the structure models in cartoon and stick representation.
