# Supplementary material for: Structural insights into human topoisomerase 3β DNA and RNA catalysis and nucleic acid gate dynamics
Source: Nat Commun. 2025 Jan 19;16:834. doi: 10.1038/s41467-025-55959-y (PMC11743793; doi:10.1038/s41467-025-55959-y)
Supplement: Supplementary file 7 — Description of Additional Supplementary Files [file 41467_2025_55959_MOESM7_ESM.pdf]

File Name: Supplementary Movie 1

Description: Structural transition between DNA and RNA binding conformations of TOP3B-TDRD3 core complex.

File Name: Supplementary Movie 2

Description: Spinning of the dimer of TOP3B-TDRD3 core complex with a DNA bubble substrate.

File Name: Supplementary Movie 3

Description: Structural transition between closed and open conformations of the TOP3B-TDRD3 core complex. The catalytic tyrosine and the residues forming the catalytic divalent cation binding motif are highlighted in the ball-and-stick representation.

File Name: Supplementary Movie 4

Description: Structural transition showcasing the closed-to-open conformations of the TOP3B-TDRD3 core complex, viewed from domain II. The catalytic tyrosine and the residues forming the catalytic divalent cation binding motif are highlighted in the ball-and-stick representation.
